# Fine-tuning the electronic structure of heavy-atom-free **BODIPY** photosensitizers for fluorescence imaging and mitochondria-targeted photodynamic therapy†

**DOI:** 10.1039/d0sc01171a

**Published:** 2020-03-17

**Authors:** Sujie Qi, Nahyun Kwon, Yubin Yim, Van-Nghia Nguyen, Juyoung Yoon

**Affiliations:** Department of Chemistry and Nanoscience, Ewha Womans University Seoul 03760 Republic of Korea jyoon@ewha.ac.kr; Institute of Research and Development, Duy Tan University Da Nang 550000 Vietnam nguyenvannghia8@duytan.edu.vn

## Abstract

Theranostics that combines both diagnosis and therapy into a single platform has recently emerged as a promising biomedical approach for cancer treatment; however, the development of efficient theranostic agents with excellent optical properties remains a challenge. Here, we report novel mitochondria-targeting **BODIPY** photosensitizers (**R-BOD**s) that possess considerable singlet oxygen generation capabilities and good fluorescence properties for imaging-guided photodynamic therapy (PDT). The incorporation of sulfur atoms into the π-conjugated skeleton of **BODIPY** along with the introduction of different functional groups at the *meso*-position of the **BODIPY** core is essential for tuning the photophysical and photosensitizing properties. Notably, the MeOPh-substituted thiophene-fused **BODIPY** (**MeO-BOD**, R = *p*-methoxyphenyl) displayed the highest singlet oxygen generation capability (*Φ*_Δ_ ≈ 0.85 in air-saturated acetonitrile) and a moderate fluorescence quantum yield (*Φ*_f_ = 17.11). Furthermore, **MeO-BOD** showed good biocompatibility, low dark toxicity and superior fluorescence imaging properties in living cells. More importantly, the PDT efficacy of mitochondria-specific anchoring of **MeO-BOD** was remarkably amplified with an extremely low half-maximal inhibitory concentration (IC_50_) value of 95 nM. We believe that the incorporation of an electron-donating group at the *meso*-position of the thiophene-fused **BODIPY** platform may be an effective approach for developing theranostic agents for precision cancer therapy.

## Abstract 2

We designed and investigated novel mitochondria-targeting heavy-atom-free BODIPY photosensitizers (R-**BODs**) that possessed considerable singlet oxygen generation abilities and good fluorescence properties for imaging-guided photodynamic therapy (PDT).

## Graphical abstract

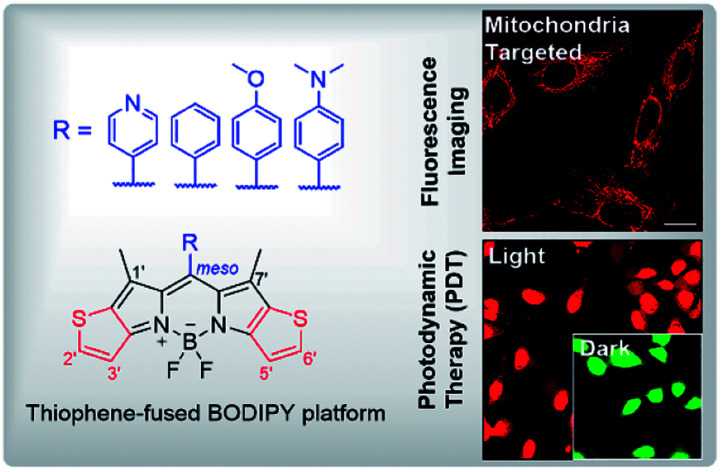

## Introduction

Photodynamic therapy (PDT) is an effective clinical treatment strategy for malignant tumors.^1^ In the PDT process, a photosensitizer (PS) is activated under light irradiation, and the excited PS subsequently interacts with molecular oxygen to generate cytotoxic reactive oxygen species (ROS), which can oxidize biomolecules resulting in cancer cell death.^2,3^ In particular, theranostics has recently been recognized as a promising medical technology that combines diagnostic and therapeutic capabilities in one dose to achieve the real-time and precise monitoring of the therapeutic effect of the drug.^4^ Moreover, PDT combined with image-guided diagnosis has attracted considerable attention owing to its prominent advantages such as high spatiotemporal selectivity, noninvasiveness, and fewer side effects.^5–7^ Therefore, the design of novel PSs that can effectively produce both fluorescence and ROS is in high demand.

To date, numerous organic dyes, such as porphyrins, phthalocyanines, cyanine, squaraine, diketopyrrolopyrrole (DPP), and boron dipyrromethane (**BODIPY**) derivatives, have been developed as theranostic agents.^7–13^ Among these, **BODIPY** dyes have attracted great interest as theranostic agents in photodynamic cancer therapy due to their excellent photochemical stability, good biocompatibility, high molar extinction coefficients, high quantum efficiencies of fluorescence and facile modification.^11–17^ At present, for obtaining remarkable PDT efficiency of **BODIPY** dyes, the most popular approach is the introduction of heavy halogen atoms (Br and I) to promote spin–orbit coupling (SOC), which enhances intersystem crossing (ISC) and improves the singlet oxygen (^1^O_2_) generation capability.^18–24^ However, the incorporation of heavy atoms increases the dark-toxicity and quench fluorescence.^25^ Thus, **BODIPY** PSs without heavy halogen atoms are preferred as theranostic agents. Recently, several approaches to enhance the ISC, such as the use of double excited states,^26,27^ spin converters,^28^ and photoinduced electron transfer (PET),^29–31^ have been implemented in the development of PSs without heavy atoms; however, the search for novel PSs is still required for PDT to reach its full potential.

Intriguingly, one alternative strategy to strengthen the ISC with an efficient triplet population is to increase the SOC while decreasing the singlet–triplet energy gaps; to do this, a thiophene moiety was introduced into the π-conjugated system of **BODIPY**,^32–35^ but the oncological applications of this approach have not been fully studied.^35^ Furthermore, the introduction of electron-donating groups at the *meso*-site of the **BODIPY** platform is also an emerging strategy for achieving efficient PSs.^36,37^ The abovementioned factors inspired us to consider designing PSs with different functional substituents incorporated at the *meso*-position of thiophene-fused **BODIPY** to achieve compounds suitable for clinical use. To the best of our knowledge, there have been no attempts to design efficient theranostic agents by taking advantage of the above two strategies.

**Scheme 1:**
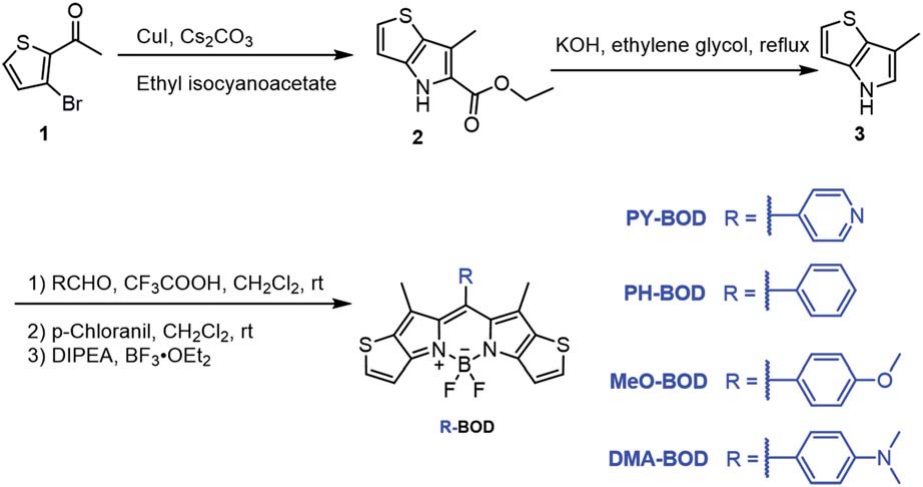
Synthetic route to the thiophene-fused **BODIPY** derivatives.

Herein, we designed and synthesized a series of heavy-atom-free thiophene-**BODIPY** derivatives by incorporating different functional groups (pyridinyl (**PY**), phenyl (**PH**), *p*-methoxyphenyl (**MeO**) and *N*,*N*-dimethylaminophenyl (**DMA**)) at the *meso*-position of the thiophene-**BODIPY** platform (Scheme 1). The experimental results revealed that the ^1^O_2_ generation ability of these thiophene-**BODIPY** derivatives through a type II process gradually increased with increasing electron-donating ability of the substituent (*Φ*_Δ_: **PY-BOD** < **PH-BOD** < **MeO-BOD**). Fortunately, **MeO-BOD** had prominent dual functions, showing both a high ^1^O_2_ quantum yield and moderate fluorescence intensity. Furthermore, the cellular experimental results showed that **MeO-BOD** could be utilized as a mitochondria-specific diagnostic agent to reinforce the PDT effect.

## Results and discussion

### Synthesis and photophysical properties

**Fig. 1:**
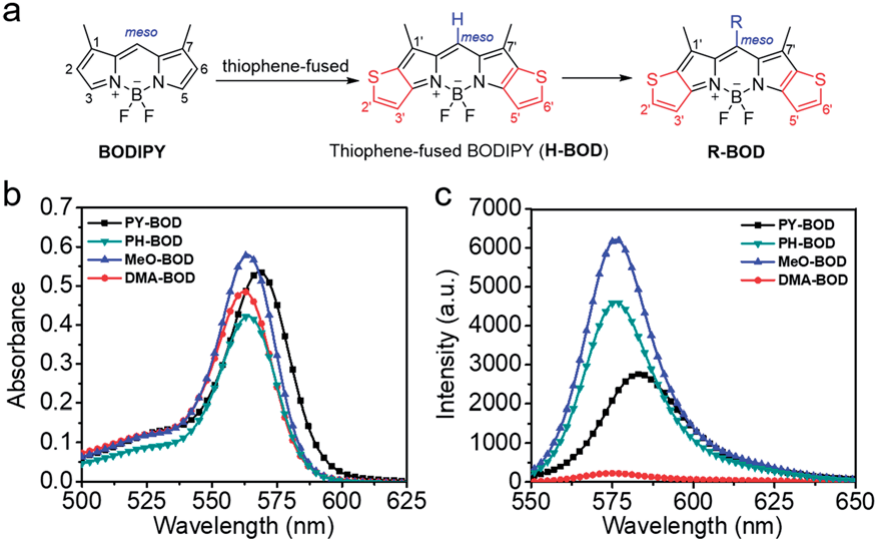
(a) Design of thiophene-fused **BODIPY** derivatives, (b) UV-Vis absorption and (c) emission spectra of **PY-BOD**, **PH-BOD**, **MeO-BOD** and **DMA-BOD** in toluene at *c* = 5.0 μM and *λ*_ex_ = 540 nm.

The different thiophene-fused **BODIPY** derivatives were synthesized through the trifluoroacetic acid-catalyzed condensation of thiophene-fused pyrroles with different benzaldehydes, followed by oxidation and complexation (Scheme 1). Detailed experimental procedures are provided in the ESI.† The structures of all these thiophene-fused **BODIPY** derivatives were fully confirmed by ^1^H and ^13^C NMR spectroscopy and high-resolution mass spectrometry (Fig. S1–S14†). UV-vis absorption and fluorescence spectra of the new thiophene-**BODIPY** derivatives were acquired (Fig. 1, S15 and S16†). These key photophysical property data are summarized in Table 1.

**Table 1:** Photophysical properties of the synthesized compounds

Compounds	*λ*_abs_ a [nm]	*ε* × 10^−3^^a^ [M^−1^ cm^−1^]	*λ*_em_ a [nm]	Stokes shift [nm]	*Φ*_f_ b (%)	*Φ*_Δ_ c
**BODIPY** d	502	120.00	508	6	70.0	≈0
**PY-BOD**	568	107.09	585	17	9.82	0.52
**PH-BOD**	564	111.60	576	12	18.83	0.69
**MeO-BOD**	564	115.78	579	15	17.11	0.85
**DMA-BOD**	562	96.99	577	15	2.50	0.04

a In toluene (5.0 × 10^−6^ M).

b Fluorescence quantum yield estimated relative to rhodamine 101 as the standard (*Φ*_f_ = 1.0 in methanol).

c Singlet oxygen quantum yield was determined with respect to rose bengal (*Φ*_Δ_ (RB) = 0.54 in ACN).

d Literature value.^19^

The absorption and fluorescence spectra of all the thiophene-fused **BODIPY** derivatives were similar in shape to those of **H-BOD**, indicating that the R substituents had little effect on the HOMO–LUMO energy gap of the thiophene-fused **BODIPY** chromophore because they are nearly orthogonal to the thiophene-fused **BODIPY** core (Fig. 1a).^32^ All the studied compounds retained high absorption molar extinction coefficients (*ε* ∼ 100 000 M^−1^ cm^−1^), and the maximum absorptions were shifted to longer wavelengths (*λ*_abs_ ∼ 565 nm) compared with that of unmodified **BODIPY**. The absorption spectra of all the thiophene-**BODIPY** derivatives showed a slight blueshift with increasing solvent polarity. This phenomenon indicates that the dipole moments of the ground state of these thiophene-**BODIPY** derivatives might be larger than those of the first singlet excited states.^32^ On the other hand, **PY-BOD**, **PH-BOD** and **MeO-BOD** exhibited moderate fluorescence intensities, while **DMA-BOD** was poorly emissive due to a PET mechanism.^38,39^ The fluorescence quantum yields of all the thiophene-**BODIPY** derivatives were evaluated in toluene (Table 1), and **PY-BOD**, **PH-BOD** and **MeO-BOD** were shown to be excellent candidates for fluorescence imaging.

### ^1^O_2_ generation ability

**Fig. 2:**
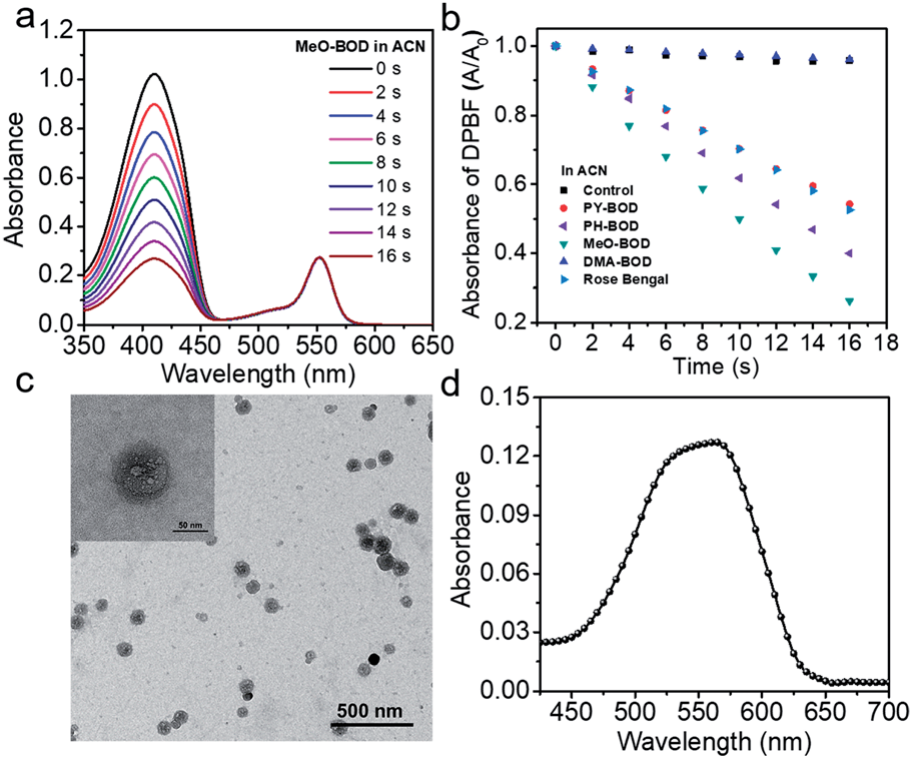
(a) Time-dependent photodegradation of DPBF with **MeO-BOD**; (b) the DPBF degradation rate curves with **PY-BOD**, **PH-BOD**, **MeO-BOD**, **DMA-BOD** and rose bengal in ACN; (c) a TEM image of **MeO-BOD** NPs (inset: high magnification TEM image); and (d) UV-Vis absorption spectrum of **MeO-BOD** (5 μM) in deionized water.

Subsequently, the singlet oxygen generation capability of **PY-BOD**, **PH-BOD**, **MeO-BOD** and **DMA-BOD** was assessed in air-saturated acetonitrile (ACN) under 560 nm irradiation. A commercial ^1^O_2_ probe, 1,3-diphenylisobenzofuran (DPBF), was used as an indicator, and rose bengal (RB, *Φ*_Δ_ = 0.54 in ACN) was used as the reference.^40^ As shown in Fig. 2a and S17,† the absorbance at 410 nm of DPBF decreased gradually in the presence of the thiophene-**BODIPY** derivatives under continuous light irradiation. According to the linear relationship of the decay curves (Fig. 2b), the ^1^O_2_ quantum yields of **PY-BOD**, **PH-BOD**, **MeO-BOD** and **DMA-BOD** were calculated to be 0.52, 0.69, 0.85 and 0.04, respectively (Table 1). The strongest ^1^O_2_ generation ability of **MeO-BOD** among all the **R-BOD**s suggested the electron donating group (MeOPh–) in the thiophene-fused **BODIPY** derivatives played an important role in enhancing the ^1^O_2_ generation. Based on these results, we propose a plausible mechanism for **MeO-BOD**. The introduction of the donating group probably favors ^1^O_2_ generation first by increasing the formation of the charge transfer (CT) state *via* photoinduced charge transfer (PCT) (^1^**BOD**-Donor → **BOD**^[*δ*−]^-Donor^[*δ*+]^). The charge recombination of the CT state further triggers the production of T_1_ of the thiophene-fused **BODIPY** derivatives (**BOD**^[*δ*−]^-Donor^[*δ*+]^ → ^3^**BOD**-Donor). Finally, ^1^O_2_ is generated by energy transfer from T_1_ to molecular oxygen (^3^**BOD**-Donor + O_2_ → **BOD**-Donor + ^1^O_2_) (Scheme S1†).^29–31,37,41,42^ Unfortunately, **DMA-BOD** showed almost no ^1^O_2_ generating ability in ACN, which implied that the electron-donating group could also lead to a PET process, which triggered the quenching of fluorescence and forbidding of non-radiative transitions such as ISC, thereby prohibiting the ^1^O_2_ generation.^43^ Thus, the ^1^O_2_ formation efficiency could be controlled by finely tuning the electronic properties of the *meso*-substituent on the thiophene-**BODIPY** platform, especially by the introduction of a suitable electron-donating group. Taken together, these results indicate that **MeO-BOD** has potential as a theranostic agent for cancer treatment.

### Cellular fluorescence imaging and subcellular colocalization

Given the above inspiring results, we attempted to investigate the fluorescence (FL) imaging and PDT efficiency of **MeO-BOD** in living cells. To this end, **MeO-BOD** nanoparticles (NPs) were prepared by adding the DMSO stock solution of **MeO-BOD** into water.^44^ The transmission electron microscopy (TEM) image in Fig. 2c indicated that the **MeO-BOD** NPs had a regular spherical morphology with a diameter of approximately 72 nm. The size distribution of the nanoparticles was determined using dynamic light scattering (DLS), which showed that the average size was 68 ± 7 nm (Fig. S18†). These particles are suitably sized for passive targeting through the enhanced permeability and retention (EPR) effect.^45^ In addition, the evident decrease in absorbance and the broad absorption spectrum and the redshift of the fluorescence spectrum of **MeO-BOD** in deionized water (DW) suggested that the formation of **MeO-BOD** NPs might be due to J-aggregation (Fig. 2d and S19b†).^46^ The **MeO-BOD** NPs could be disassembled and the instinctive fluorescence peak of **MeO-BOD** could be restored in the presence of FBS (10%) in DW (Fig. S19†).

**Fig. 3:**
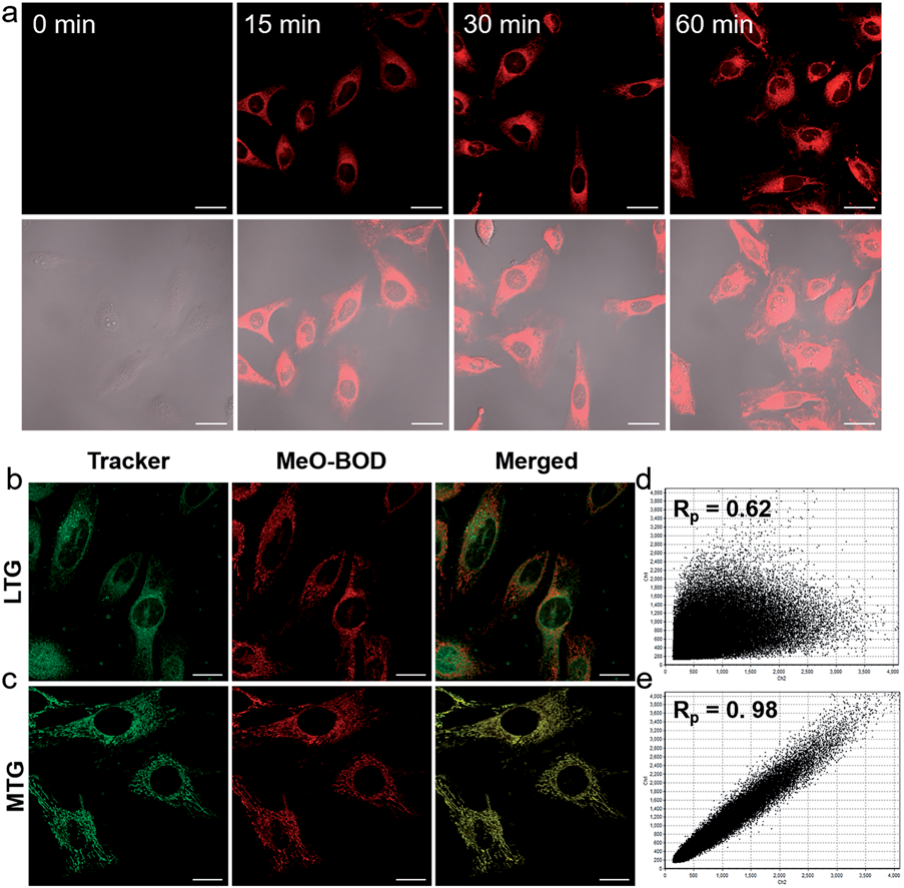
(a) Confocal laser scanning microscopy images of HeLa cells incubated with **MeO-BOD** (1.0 μM) for different times (0, 15, 30 and 60 min); scale bar: 30 μm. Confocal laser scanning microscopy colocalization fluorescence images of **MeO-BOD** (1.0 μM) with (b) LTG and (c) MTG (500 nM) in HeLa cells, respectively. The fluorescence intensity correlation of **MeO-BOD** with (d) LTG and (e) MTG, respectively. *R*_p_ is Pearson's coefficient; scale bar: 20 μm.

Then, we further explored the cellular uptake of **MeO-BOD** in HeLa cells by using confocal laser scanning microscopy. As illustrated in Fig. 3a, **MeO-BOD** could be rapidly internalized by living cells, and the FL images showed strong emission in the cell cytoplasm. Therefore, **MeO-BOD** could be employed as an imaging-guided PDT agent. Furthermore, to test the main organelle locations of **MeO-BOD**, we co-stained HeLa cells with **MeO-BOD** and commercial MitoTracker Green (MTG) or LysoTracker Green (LTG). The colocalization experiments indicated that **MeO-BOD** were mainly localized in mitochondria, as indicated by the high Pearson's coefficient (0.98), instead of the lysosomes (Pearson's coefficient 0.62) (Fig. 3b–e). In addition, the time-dependent colocalization fluorescence imaging of **MeO-BOD** with MTG in HeLa cells was performed. As shown in Fig. S20,†**MeO-BOD** almost internalize into the mitochondria of HeLa cells after 1 h. Recent reports have demonstrated that the **BODIPY** dye itself could localize into the mitochondria of cells due to its low electron density character.^47^ The unmodified **BODIPY** platform has a low electron density (+*δ*) character. Also, the incorporation of sulfur atoms into the π-conjugated skeleton of **BODIPY** perhaps led to more reduction of electron density of the **BODIPY** core,^48^ which might facilitate the mitochondrial accumulation of **MeO-BOD**. Subcellular organelles are indispensable in maintaining cellular biological function.^49^ In particular, the generation of ^1^O_2_ in mitochondria can induce direct dysfunction and trigger cell apoptosis. Thus, mitochondria-targeted theranostic agents could maximize cancer treatment efficiency.

### *In vitro* PDT efficacy evaluation

**Fig. 4:**
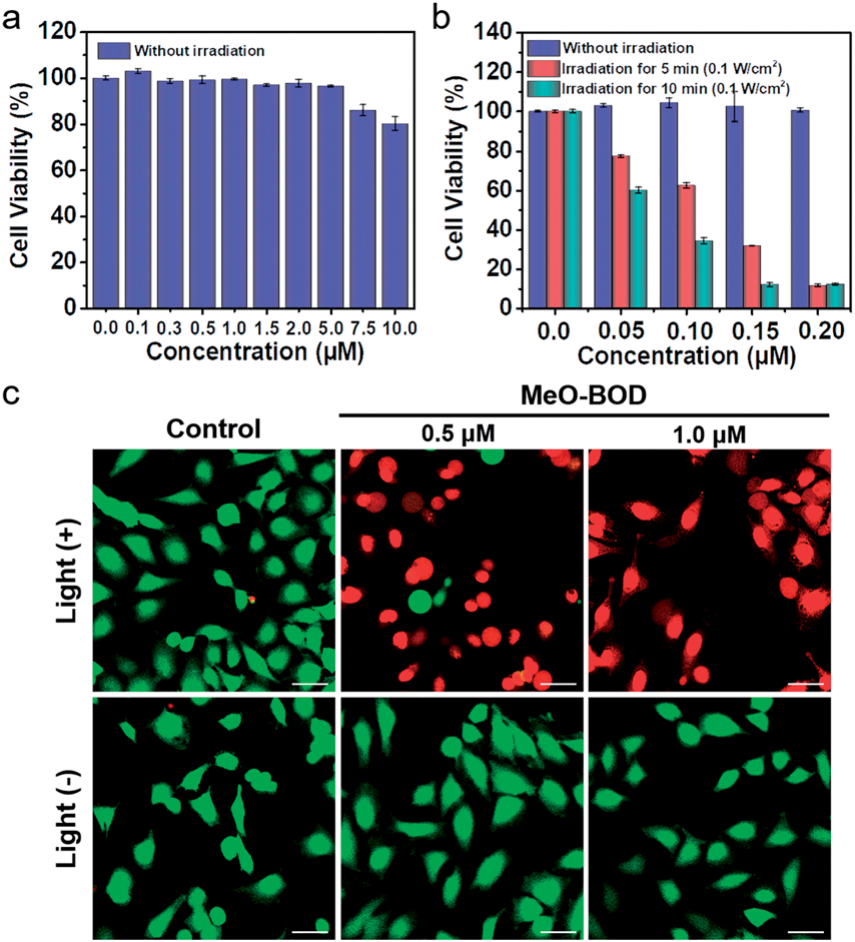
Concentration-dependent changes in the cell viability of HeLa cells treated with **MeO-BOD** using a typical MTT assay (a) in the dark and (b) under light irradiation. Cells were irradiated with 560 nm light (0.1 W cm^−2^, 5 and 10 min). (c) Fluorescence images of calcein AM/PI-stained HeLa cells after preincubation with **MeO-BOD** (0.5 and 1.0 μM) for 1 h and irradiation with 560 nm light (0.1 W cm^−2^, 10 min). Scale bar: 40 μm.

To demonstrate the PDT efficacy of **MeO-BOD** in living cells, we first tested its cytotoxicity by the methyl thiazolyltetrazolium (MTT) assay in HeLa cells. As indicated in Fig. 4a, **MeO-BOD** had negligible cytotoxicity in the dark, revealing its excellent biocompatibility *in vitro*. Under 560 nm light irradiation (0.1 W cm^−2^, 5 or 10 min), the viability of HeLa cells gradually decreased with the increasing concentration of **MeO-BOD**, and the growth inhibition ratio reached ∼88% even at a very low concentration of 0.15 μM (Fig. 4b). The half-maximal inhibitory concentration (IC_50_) of **MeO-BOD** for HeLa cells was as low as 95 nM under 560 nm light irradiation (0.1 W cm^−2^, 10 min). The extremely low IC_50_ of **MeO-BOD** could be attributed to the high ^1^O_2_ quantum yield and efficient mitochondria-specific ROS generation upon light irradiation. To clarify the cytotoxicity in the PDT process, we tracked the morphological variations of HeLa cells in the presence of **MeO-BOD** under 559 nm laser irradiation using confocal laser scanning microscopy. As described in Fig. S21,† with increasing irradiation time (0–10 min), the morphology of the HeLa cells preincubated with **MeO-BOD** obviously changed; gradual thinning of the cell membrane and the formation of numerous blebs (red line) were observed. In contrast, the cells not exposed to **MeO-BOD** exhibited no appreciable morphological changes under the same laser irradiation.

Additionally, to intuitively establish the PDT efficacy of **MeO-BOD** (0.5 and 1.0 μM), live/dead cell co-staining of HeLa cells with calcein AM (green, live cells) and PI (red, dead cells) was performed to visualize the cell viability (Fig. 4c). The control group showed strong green fluorescence for live cells, which also verified the low dark toxicity and good biocompatibility of **MeO-BOD**. In comparison, almost all the HeLa cells treated with **MeO-BOD** were killed, and intense red fluorescence was observed. All these results confirmed that **MeO-BOD** could be employed as a theranostic agent for cancer treatment *in vitro*.

### Apoptosis mechanism of **MeO-BOD**-mediated PDT

**Fig. 5:**
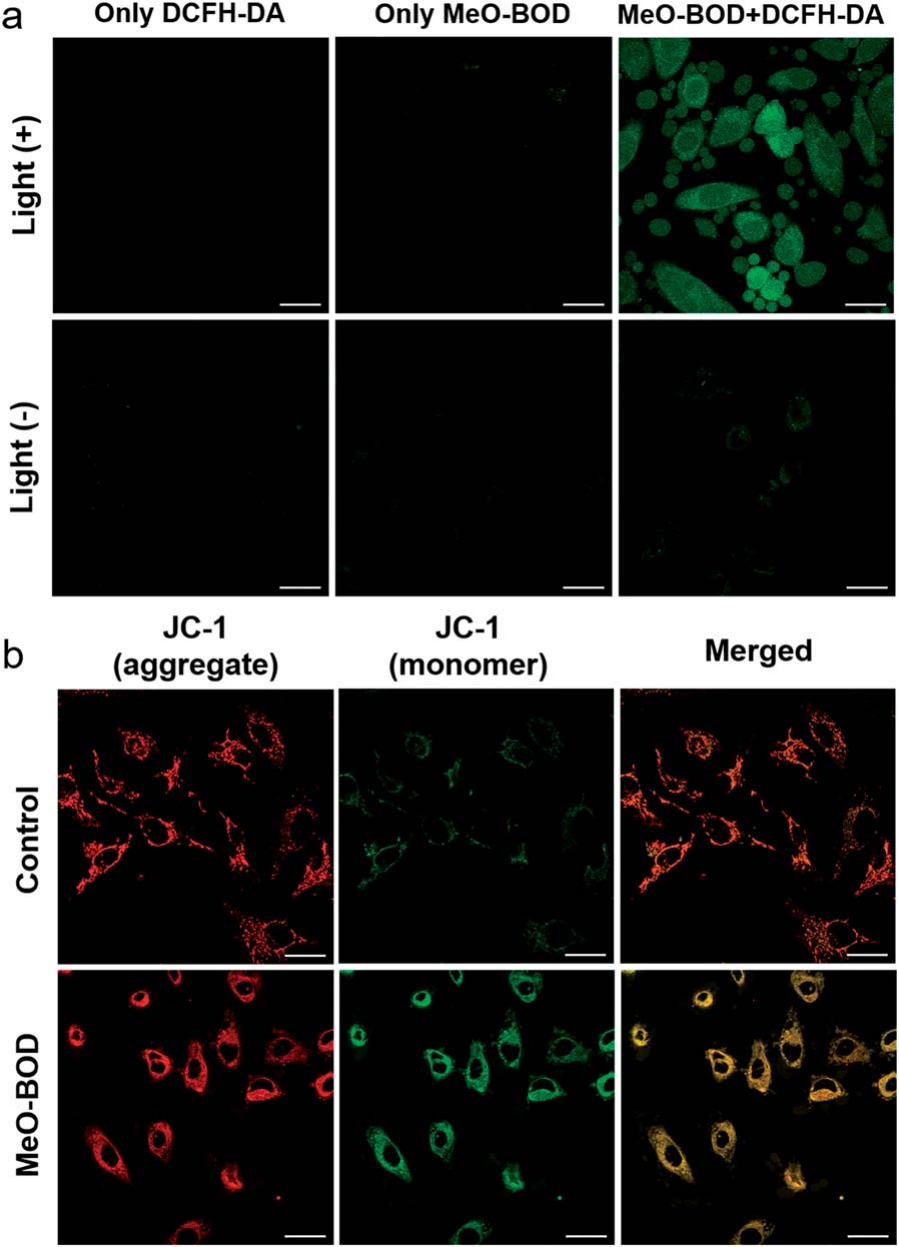
(a) Fluorescence images of ROS generation in HeLa cells after incubation with **MeO-BOD** (1.0 μM) for 1 h using DCFH-DA as an indicator (10 μM). The green fluorescence indicates that DCFH-DA is oxidized to DCF. (b) Fluorescence images of the mitochondrial membrane potential of HeLa cells after incubation without or with **MeO-BOD** (1.0 μM) using JC-1 as the indicator (2 μM), *λ*_ex_ = 473 nm, red channel for aggregates (healthy cells): 575–675 nm, and green channel for monomers (apoptotic cells): 490–540 nm. Samples were irradiated with 560 nm light (0.1 W cm^−2^, 10 min). Scale bar: 30 μm.

Finally, to elucidate the potential therapeutic mechanism at the cellular level, we further evaluated the cellular ^1^O_2_ generation capability of **MeO-BOD** by using 2,7-dichlorodihydrofluorescein diacetate (DCFH-DA) as the ^1^O_2_ indicator in HeLa cells (Fig. 5a). The HeLa cells treated with only DCFH-DA and only **MeO-BOD** showed almost no fluorescence, whereas the group pretreated with **MeO-BOD** before light irradiation and then incubated with DCFH-DA showed apparent green fluorescence from oxidized 2,7-dichlorofluorescein (DCF). These experimental results validated the ^1^O_2_ generation ability of **MeO-BOD** in living cells. As mentioned above, the production of ^1^O_2_ in mitochondria causes mitochondrial destruction, resulting in cell apoptosis. The reduction of mitochondrial membrane potential (MMP) is a crucial signal of mitochondrial damage. Hence, the MMP changes were monitored by using confocal fluorescence images using JC-1 dye, as its fluorescence color changed between its aggregates (red, high MMP) and monomers (green, low MMP). The untreated control group under 560 nm light irradiation (0.1 W cm^−2^ for 10 min) displayed strong red emission and weak green emission, indicating that the cells were healthy with a high MMP. By comparison, the cells treated with **MeO-BOD** suffered from depolarization of the mitochondrial membrane, as demonstrated by the increase of green fluorescence intensity (Fig. 5b). These observations further suggested that the cell death was induced by a mitochondria-associated pathway.

## Conclusions

We synthesized thiophene-fused **BODIPY** analogues with different functional groups at the *meso*-position of the **BODIPY** core as mitochondria-targeted theranostic agents for imaging and PDT. The varying R-substituents had little effect on the absorption and emission maxima. **PY-BOD**, **PH-BOD** and **MeO-BOD** exhibited moderate fluorescence quantum yields, while that of **DMA-BOD** was much lower due to a PET process. Among the analogues, **MeO-BOD** exhibited excellent dual functionality, both considerable ^1^O_2_ generation ability and high brightness. The cell experiments manifested that **MeO-BOD** offered many advantages: good biocompatibility, mitochondria-specific fluorescence imaging, and a very low IC_50_ value (≈95 nM). Therefore, **MeO-BOD** could be employed as an imaging-guided PDT agent for cancer treatment. In addition, the apoptosis mechanism of light-induced PDT might be a result of ROS-induced damage to mitochondria, which was demonstrated by detecting the changes of the mitochondrial membrane potential. Briefly, finely tuning the electronic structure of the substituent at the *meso*-site of a heavy-atom-free thiophene-fused **BODIPY** core is a promising strategy for developing highly efficient theranostic agents with minimal side effects to accomplish the integration of diagnosis and therapy.

## Conflicts of interest

There are no conflicts to declare.

## Supplementary Material

SC-011-D0SC01171A-s001
