# Supplementary material for: Fine-tuning the electronic structure of heavy-atom-free BODIPY photosensitizers for fluorescence imaging and mitochondria-targeted photodynamic therapy
Source: Chem Sci. 2020 Mar 17;11(25):6479–84. doi: 10.1039/d0sc01171a (PMC8152625; doi:10.1039/d0sc01171a)
Supplement: SC-011-D0SC01171A-s001 [file SC-011-D0SC01171A-s001.pdf]

Electronic Supplementary Material (ESI) for Chemical Science.
This journal is © The Royal Society of Chemistry 2020

Supporting Information

Fine-Tuning the Electronic Structure of Heavy-Atom-Free BODIPY Photosensitizers for Fluorescence Imaging and Mitochondria-Targeted Photodynamic Therapy

Sujie Qi,[a] Nahyun Kwon,[a] Yubin Yim,[a] Van-Nghia Nguyen,*[ab] and Juyoung Yoon*[a]

[a] *Department of Chemistry and Nanoscience, Ewha Womans University, Seoul 03760, Republic of Korea. E-mail: jyoon@ewha.ac.kr*

[b] *Institute of Research and Development, Duy Tan University, Da Nang 550000, Vietnam. E-mail: nguyenvannghia8@duytan.edu.vn*

Table of contents

1. Experimental details...………………………….……………………….…………S3-S6

2. Compound synthesis…………………...……………….…………..……..….…… S7-S9

3. NMR spectra and HR-MS…………………………….……………………..…..… S10-S16

4. Optical properties and biological studies ………………………………..…. S17-S22

5.Supporting references……………………………….…………...………...……S22

1. Experimental Details

Reagents and instruments. All chemicals were purchased from Sigma and used without further purification. DCFH-DA, MitoTracker Green FM, and LysoTracker Green DND-26 were purchased from Thermo (invitrogen). Calcein-AM and PI was purchased from Sigma. Deuterated solvents from Cambridge Isotope Laboratories were used. All the ^{1}H-NMR and ^{13}C-NMR spectra were performed with a Bruker AM 300 (300.13 MHz for ^{1}H, 75.48 MHz for ^{13}C) spectrometer. A Jeol JMS 700 high-resolution mass spectrometer was used to acquire fast atom bombardment (FAB) mass spectra at the Korea Basic Science Institute (Daegu). UV-Visible absorption and fluorescence spectra were recorded on a Thermo Scientific Evolution 201 UV-Visible spectrophotometer and JASCO spectrofluorometer FP-8500, respectively. Dynamic light scattering (DLS) was measured using a Nano-ZS (Malvern). TEM images were recorded on a JEOL-2100F electron microscope operating at 200 kV. Confocal laser scanning microscope images were performed on Olympus Fluoview FV1200 confocal laser scanning microscope.

Calculation of fluorescence quantum yield. The fluorescence quantum yield (Φ) of the BODIPY derivatives was determined according to following equation: $\Phi_x = \Phi_{st}\cdot(I_x/I_{st})\cdot(A_{st}/A_x)\cdot(\eta_x^2/\eta_{st}^2)$, where Φ is the quantum yield, I is the area under the fluorescence spectra, A is the absorbance at the excitation wavelength, and η is the refractive index of the used solvent ($n_{MeOH} = 1.3284$ and $n_{toluene} = 1.4961$). x and st stand for BODIPY derivatives and standard, respectively. Rhodamine 101 ($\Phi_{st} = 1.0$ in methanol) was chosen as the standard for measuring the quantum yield.[1]

Singlet oxygen quantum yield calculations. The relative singlet oxygen quantum yields were determined using rose bengal (RB, $\Phi_{\Delta} = 0.54$ in ACN)[2] as a reference for all the thiophene-fused **BODIPY** derivatives (**PY-BOD**, **PH-BOD**, **MeO-BOD** and **DMA-BOD**). 1,3-Diphenylisobenzofuran (DPBF, as singlet oxygen probe, abs ~ 1.00) and photosensitizers (abs ~ 0.2) were added in a cuvette containing air-saturated organic solvents and the solutions were kept in dark until the absorbance reading was stable, followed by continuous light irradiation. The slopes of absorbance of DPBF at 410 nm versus irradiation time was recorded to obtain the $^1O_2$ generation ability. The measurements were performed using ~560 nm light that was wavelength-selected from a 500 W halogen lamp by using an optical bandpass filter.

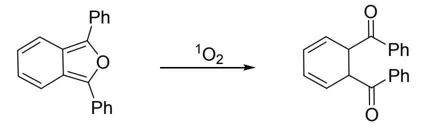

The singlet oxygen ($^1O_2$) quantum yields of the thiophene-fused **BODIPY** derivatives were determined by the equation, $\Phi_{\Delta} = \Phi_{\Delta}{}^{st} \times (S/S_{st}) \times (F_{st}/F) \times (PF_{st}/PF)$, where S is the slope of the change in absorbance of DPBF at the absorbance maxima with the irradiation time. F is the absorption correction factor, which is given as $F = 1\text{-}10^{-A}$, and PF is absorbed photonic flux (μEinstein dm^{-3} s^{-1}).

Cell culture and imaging. HeLa (human cervix adenocarcinoma) cells were obtained from Korean Cell Line Bank (Seoul, Korea). Cells were grown in Minimum Essential Medium Eagle (MEM) supplemented with 10 % fetal bovine serum, 100 U/ml penicillin and 100 U/ml streptomycin and kept in 5 % CO_2 at 37 ℃. Cells were seeded in a 35-mm glass bottomed dishes at a density of 2×10^5 cells per dish in culture media. After overnight culture, HeLa

cells were used for cell imaging. Particularly, for real-time cellular uptake assays, HeLa cells were stained with 1 μM **MeO-BOD** for various time (0, 15, 30 and 60 min). After washing with DPBS, the red fluorescence imaging was captured by confocal laser scanning microscopy (CLSM). The excitation wavelength was 559 nm, and collection wavelength was from 575 nm to 675 nm.

Subcellular colocalization assay. HeLa cells were plated onto 35 mm confocal dishes and incubated for 24 h. After incubated with 1 μM **MeO-BOD** for 1 h at 37 ℃ under 5% CO_2, the cells were further stained by LysoTracker Green DND 26 or MitoTracker Green FM (500 nM). Cells imaging were performed with laser confocal microscopy. The excitation wavelength for **MeO-BOD** was 559 nm, while the excitation wavelength for LysoTracker Green DND 26 and MitoTracker Green FM was 473 nm. The emission wavelengths were collected from 575 to 675 nm for **MeO-BOD**, 490 to 540 nm for LysoTracker Green and Mito Tracker Green.

Cytotoxicity assays. HeLa cells were seeded at 10000 cells per well in a 96-well plate with 100 μL culture media, followed by incubation under 5 % CO_2 at 37 ℃ for 24 h. **MeO-BOD** (100 μL) with varying concentrations were added into each wells, and cultured for another 24 h. After that, the cell culture media was replaced with 100 μL fresh medium. Then halogen lamp 560 nm light (0.1 W/cm^2) was employed for different irradiation time (0, 5, 10 min), and cells continued to be incubated for 24 h. Then, 10 μL MTT solutions (5 mg/mL) and 10 μL fresh MEM were added to each well for 4 h, the medium was removed out carefully, and 100 μL DMSO was added to each well to dissolve the produced blue formazan. Finally, the absorbances were read at OD 650 nm with a Spectramax Microwell plate reader. The cell viability was calculated by the following equation: Cell viability (%) = ($OD_s - OD_{blank}/OD_{control}$

− OD_{blank}) × 100%. The viability of no sample treatment cells was calculated as 100 % live. Data were represented as the mean and standard deviation (SD) of three independent experiments.

Dead/Live cell co-staining. The Calcein AM and propidium iodide (PI) are used for simultaneous staining the living and dead cells, respectively. Calcein AM is no fluorescence, but after entering the living cells, the acetoxymethyl (AM) part is hydrolyzed by esterase to form a strong green fluorescence. On the other hand, PI is a nucleic acid staining dye and can't enter living cells, but it can intercalate with double helix structure of DNA to produce a strong red fluorescence after passing through the damaged cell wall of dead cells. HeLa cells were preincubated without or with **MeO-BOD** (0.5 and 1 μM) for 1 h. After washing with DPBS, the HeLa cells were irradiated with halogen lamp 560 nm light (0.1 W/cm^2) for 10 min and then simultaneous stained with 2 μM Calcein AM and 4 μM propidium iodide (PI) for 30 min. After washing with DPBS, fluorescence images were acquired by confocal laser scanning microscopy. The group without treatment of **MeO-BOD** was as a control. The excitation wavelength was 473 nm. The green fluorescence and red fluorescence were collected 490-540 nm for Calcein AM and 575-675 nm for PI.

Intracellular $^1O_2$ detection. 2,7-dichlorofluorescin diacetate (DCFH-DA) was used as the intracellular $^1O_2$ indicator, which can be converted to DCF and emit bright green fluorescence in the presence of $^1O_2$.[3] HeLa cells were preincubated with 1 μM **MeO-BOD** for 1 h, and then were stained with 10 μM DCFH-DA for another 30 min. After being washed with DPBS, HeLa cells were followed by irradiation with halogen lamp 560 nm light (0.1 W/cm^2) for 10 min. Then, fluorescence images were acquired by confocal laser scanning microscopy to evaluate

the intracellular $^1O_2$ generation. The excitation wavelength was 473 nm and emission wavelengths were collected 490-540 nm for DCF.

Mitochondrial membrane potential assay. HeLa cells were seeded and cultured in 35 mm confocal dishes for 24 h. 1 μM **MeO-BOD** were then added into the medium and incubated with cells at 37 ℃ under 5% CO_2 for 1 h. Subsequently, the cells were exposed to 560 nm light irradiation (0.1 W/cm^2, 10 min) and stained by JC-1 (2 μM). As control, the cells were loaded with JC-1 alone with light irradiation.

2. Compound synthesis

Compound 2: A mixture of **1** (1020 mg, 5 mmol), CuI (95 mg, 0.5 mmol), and Cs_2CO_3 (3.25 g, 10 mmol) in DMSO (3 mL) was stirred 30 min at room temperature under N_2 atmosphere before being added ethyl isocyanoacetate (622 mg, 5.5 mmol) dropwise. After 4 h of stirring at 50 °C, the reaction mixture was extracted with CH_2Cl_2. The organic layer was washed with brine twice, and then the organic layer was dried over $MgSO_4$ and filtered. The filtrate was condensed with evaporation, and the crude product was purified by column chromatography using a mixture eluent hexane and ethyl acetate (v/v = 10:1) gave compound **2** as a white solid (559 mg, 53%). ^{1}H NMR (300 MHz, $CDCl_3$) δ 8.85 (s, 1H), 7.31 (d, *J* = 5.3 Hz, 1H), 6.91 (d, *J* = 5.3 Hz, 1H), 4.37 (q, *J* = 7.1 Hz, 2H), 2.53 (s, 3H), 1.40 (t, *J* = 7.1 Hz, 4H).

Compound **3**: The compound **2** (837 mg, 4 mmol) and potassium hydroxide (1.58 mg, 24 mmol) were suspended in ethylene glycol and heated to reflux for 4 h under dark and N2 atmosphere. After cooling to room temperature, the reaction mixture was put into cold water and extracted with CH_2Cl_2. The organic layer was dried over $MgSO_4$ and filtered. The filtrate was condensed with evaporation, and the crude product was purified by column chromatography using a mixed eluent (hexane and ethyl acetate = 50:1) gave compound **3** as a pale-yellow oil (310 mg, 56%). ^{1}H NMR (300 MHz, $CDCl_3$) δ 7.98 (bs, 1H), 7.08 (dd, *J* = 5.2, 1.3 Hz, 1H), 6.93 (d, *J* = 5.2 Hz, 1H), 6.77 (dq, *J* = 2.2, 1.1 Hz, 1H), 2.26 (d, *J* = 1.0 Hz, 3H).

Thiophene-fused **BODIPY** dyes: The pyrrole derivative **3** (2 mmol, 2 eq) and corresponding aromatic aldehyde (1 mmol, 1 eq) were dissolved in dried CH_2Cl_2 (200 mL) and trifluoroacetic acid (6 drops) were added. The mixture was stirred for 18 h at room temperature. A solution of p-Chloranil (1.2 eq) in CH_2Cl_2 (50 mL) was added in one portion and the dark purple mixture

was stirred for 30 min. Diisopropylethylamine (DIPEA, 18 eq) was added and the mixture was stirred for 30 min followed by a slow addition of $BF_3 \cdot Et_2O$ (18 eq). The mixture was stirred for 12 h, water (100 mL) was added and stirring was continued for 1 h. The reaction mixture was transferred to a separatory funnel and washed with portions of water. The organic layer was dried over $MgSO_4$ and the solvents were removed under reduced pressure. The crude product was purified by column chromatography using a mixed eluent (hexane and CH_2Cl_2) gave thiophene-fused **BODIPY** dyes as purple colored solid.

PY-BOD: Yield: 17.5%. ^1H NMR (300 MHz, $CDCl_3$) δ 8.87 (d, J = 5.6 Hz, 2H), 7.65 (d, J = 5.3 Hz, 2H), 7.42 (dd, J = 4.4, 1.6 Hz, 2H), 7.15 (d, J = 5.3 Hz, 2H), 1.61 (s, 6H). ^{13}C NMR (75 MHz, $CDCl_3$) δ 157.99, 151.10, 143.10, 141.75, 141.48, 136.82, 134.63, 133.68, 123.84, 114.48, 14.61. HRMS (ESI), calcd for ($C_{20}H_{14}BF_2N_3NaS_2^+$): m/z $[M+Na]^+$: 432.0588; found: m/z 432.0589.

PH-BOD: Yield: 7.1%. ^1H NMR (300 MHz, $CDCl_3$) δ 7.61 (d, J = 5.3 Hz, 2H), 7.57 (dd, J = 4.2, 2.4 Hz, 2H), 7.38 (dd, J = 6.6, 3.0 Hz, 2H), 7.16 (d, J = 5.3 Hz, 2H), 1.58 (s, 6H). ^{13}C NMR (75 MHz, $CDCl_3$) δ 157.06, 145.72, 143.23, 140.32, 137.58, 134.04, 129.60, 129.45, 128.20, 118.85, 114.19, 14.02. HRMS (FAB^+), calcd for (: $C_{21}H_{15}BF_2N_2S_2^+$): m/z $[M]^+$: 408.0738; found: m/z 408.0739.

MeO-BOD: Yield: 7.1%. ^1H NMR (300 MHz, DMSO) δ 8.08 (d, J = 5.3 Hz, 1H), 7.43 (d, J = 8.6 Hz, 1H), 7.19 (d, J = 8.7 Hz, 1H), 7.07 (d, J = 5.3 Hz, 1H), 3.87 (s, 2H), 1.63 (s, 3H). ^{13}C NMR (75 MHz, $CDCl_3$) δ 160.76, 157.07, 146.08, 140.27, 138.11, 134.13, 133.75, 129.68, 126.12, 114.98, 114.33, 55.55, 14.40. HRMS (FAB^+), calcd for ($C_{22}H_{17}BF_2N_2OS_2^+$): m/z [M]+: 438.0843; found: m/z 438.0846.

DMA-BOD: Yield: 8.7%. ^{1}H NMR (300 MHz, $CDCl_3$) δ 7.58 (d, *J* = 5.3 Hz, 2H), 7.20 – 7.10 (m, 4H), 6.89 – 6.73 (m, 2H), 3.07 (s, 6H), 1.71 (s, 6H). ^{13}C NMR (75 MHz, $CDCl_3$) δ 156.65, 151.18, 147.72, 139.62, 138.41, 134.13, 133.50, 129.39, 121.02, 114.35, 112.47, 40.42, 14.59. HRMS (ESI), calcd for ($C_{23}H_{20}BF_2N_3NaS_2^+$): m/z $[M+Na]^+$: 474.1057; found: m/z 474.1059.

3. NMR spectra and HR-MS

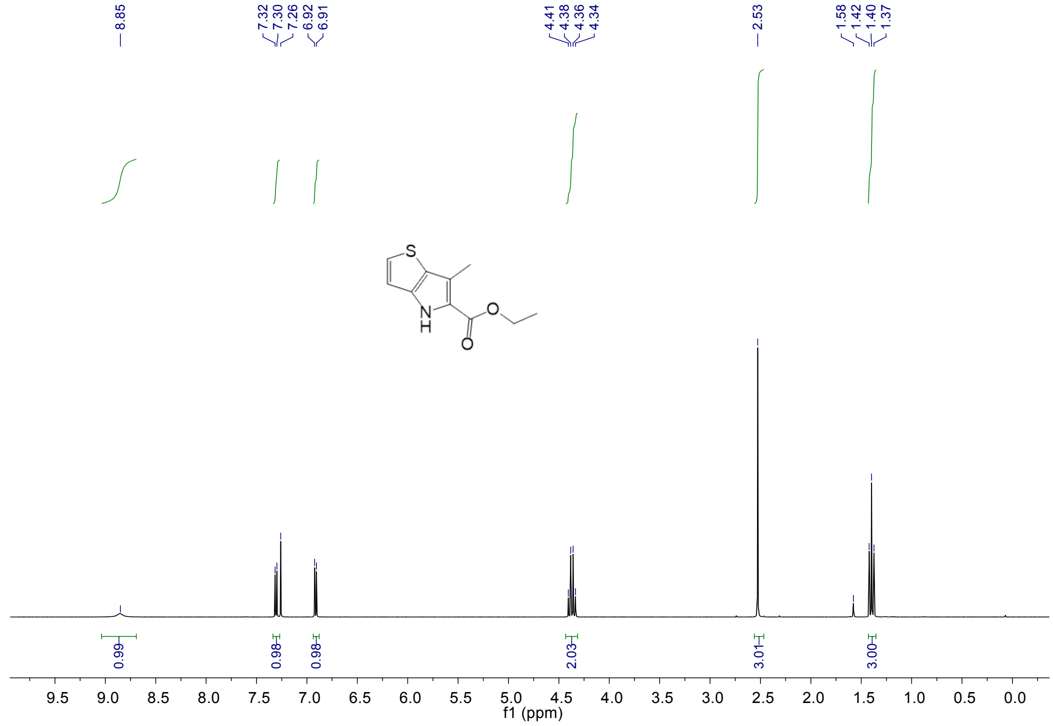

Fig. S1 ^{1}H NMR spectrum ($CDCl_3$) of compound **1**.

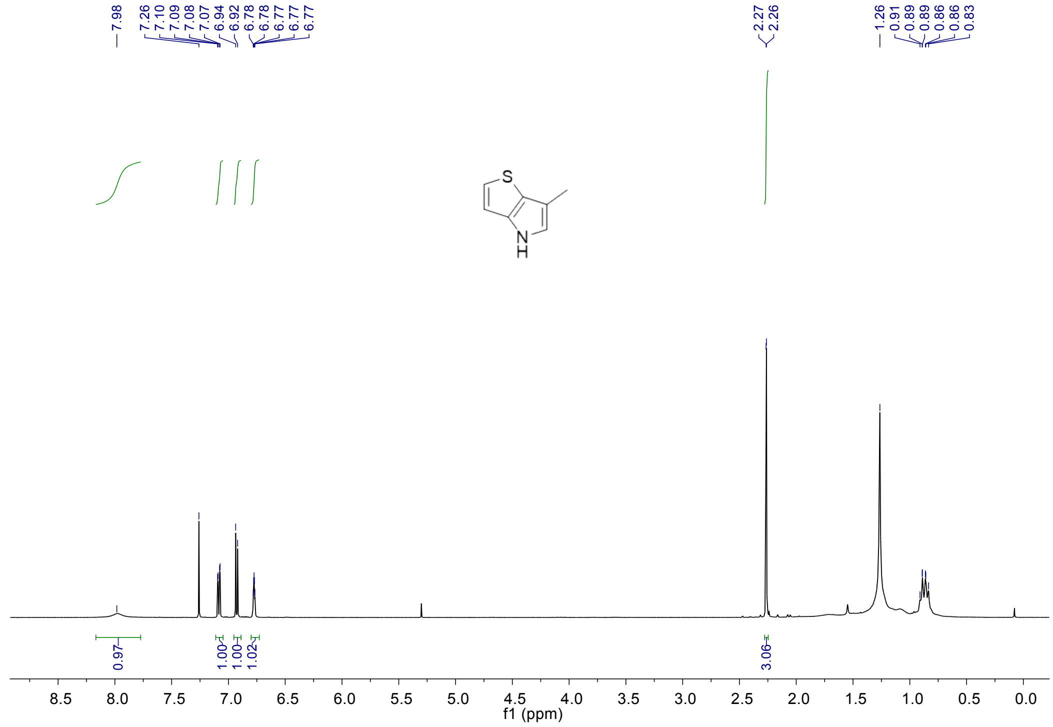

Fig. S2 ^{1}H NMR (300 MHz, $CDCl_3$) of compound **2**.

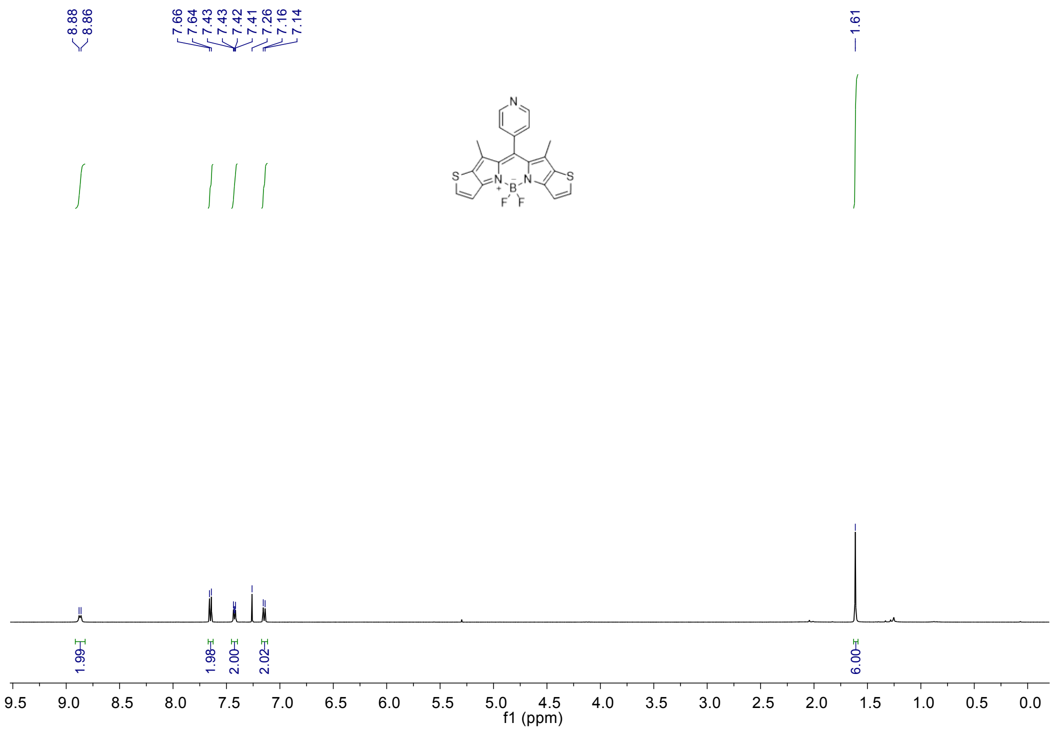

Fig. S3 ^{1}H NMR (300 MHz, $CDCl_3$) of **PY-BOD**.

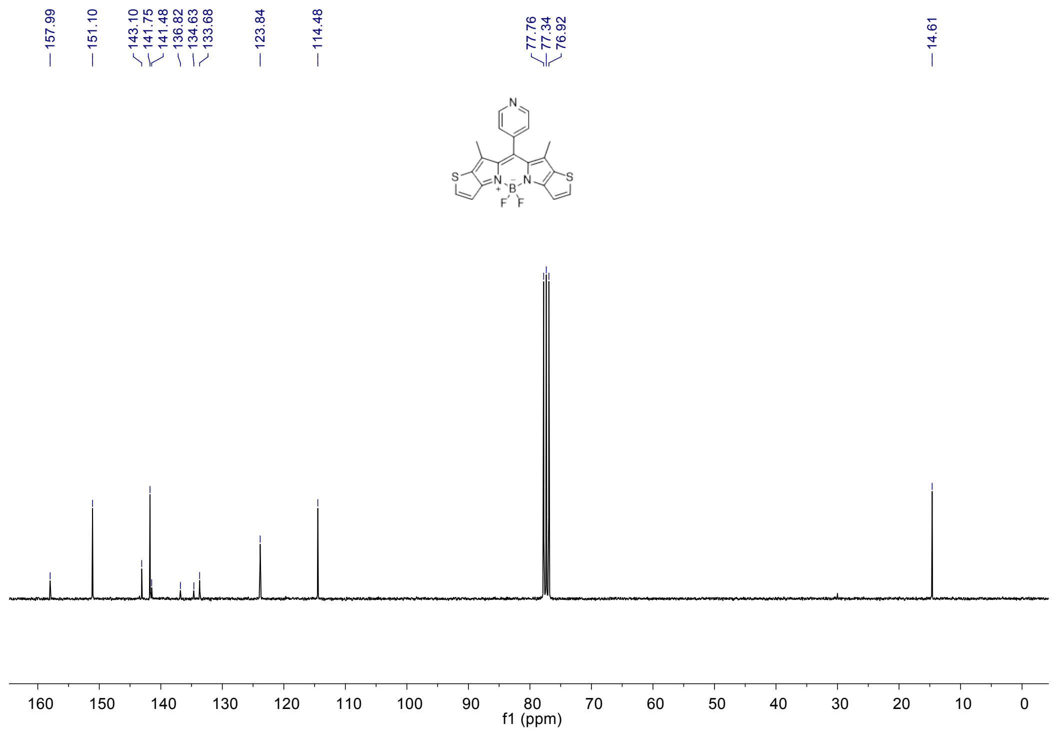

Fig. S4 ^{13}C NMR (75 MHz, $CDCl_3$) of **PY-BOD**.

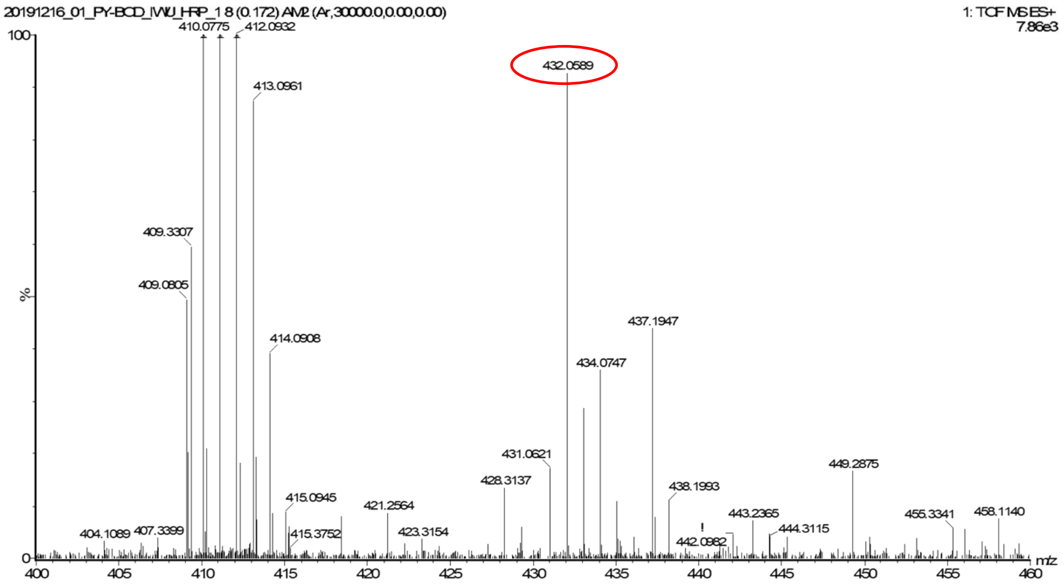

Fig. S5 High resolution mass spectrum of **PY-BOD**.

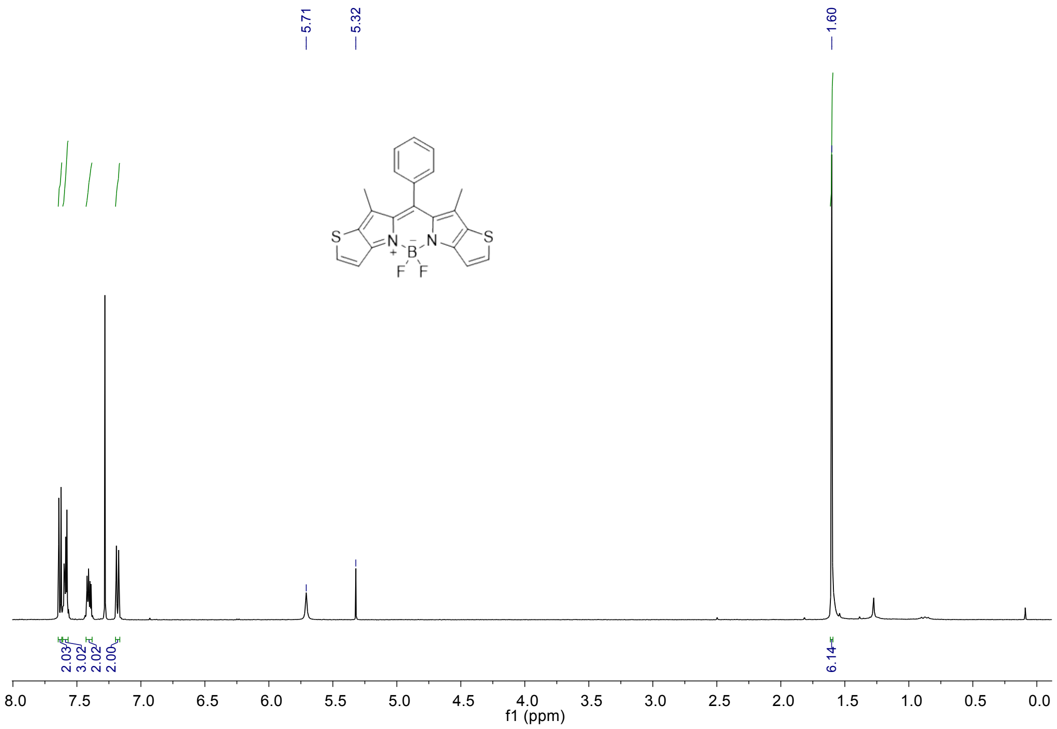

Fig. S6 ^{1}H NMR (300 MHz, $CDCl_3$) of **PH-BOD**.

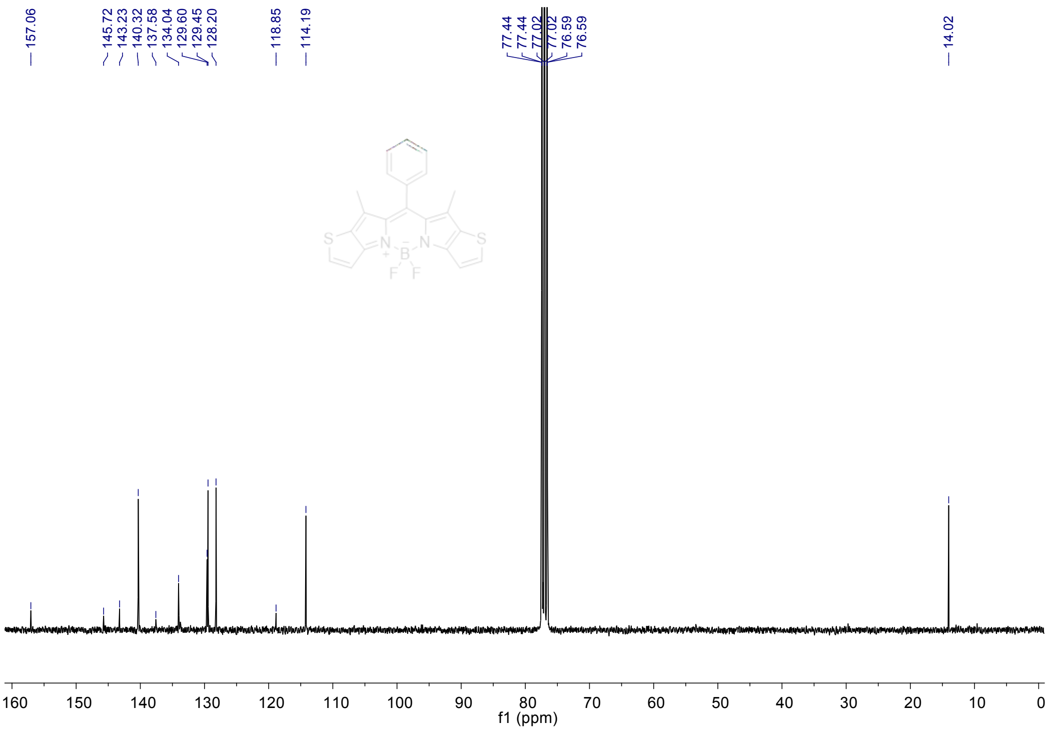

Fig. S7 ^{13}C NMR (75 MHz, $CDCl_3$) of **PH-BOD**.

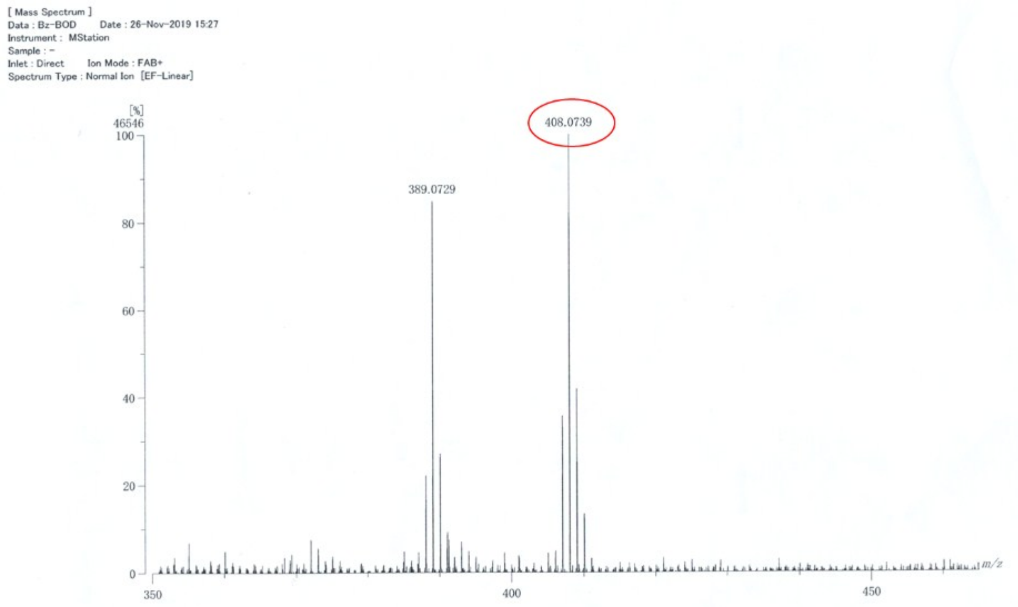

Fig. S8 High resolution mass spectrum of **PH-BOD**.

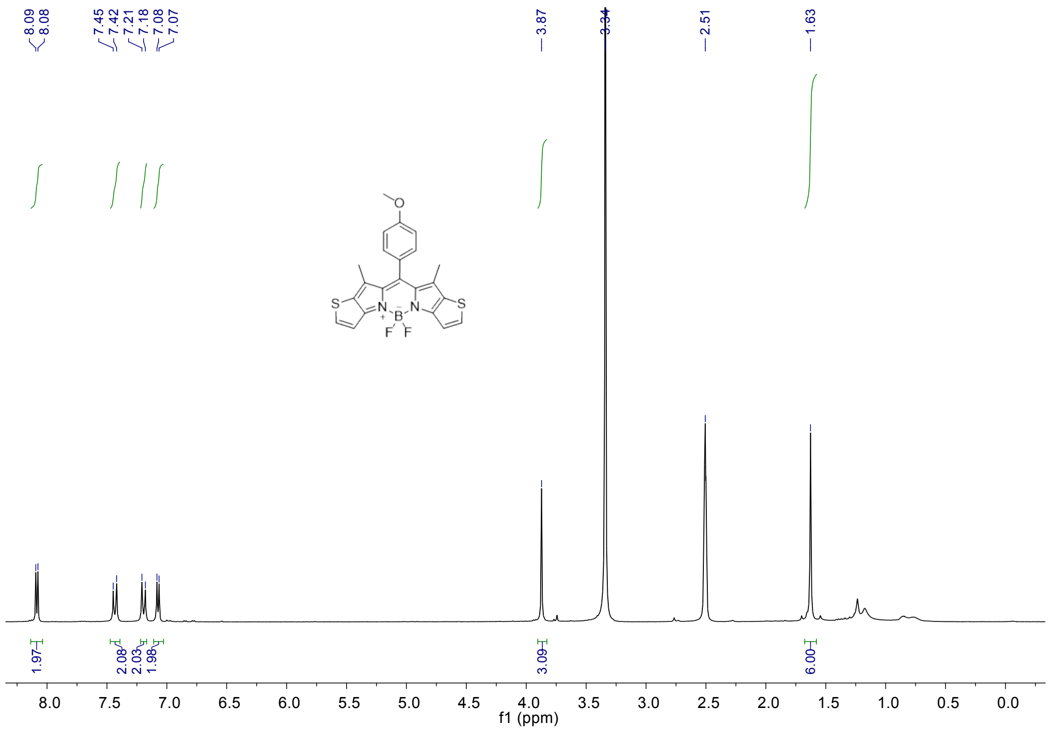

Fig. S9 ^{1}H NMR (300 MHz, DMSO) of **MeO-BOD**.

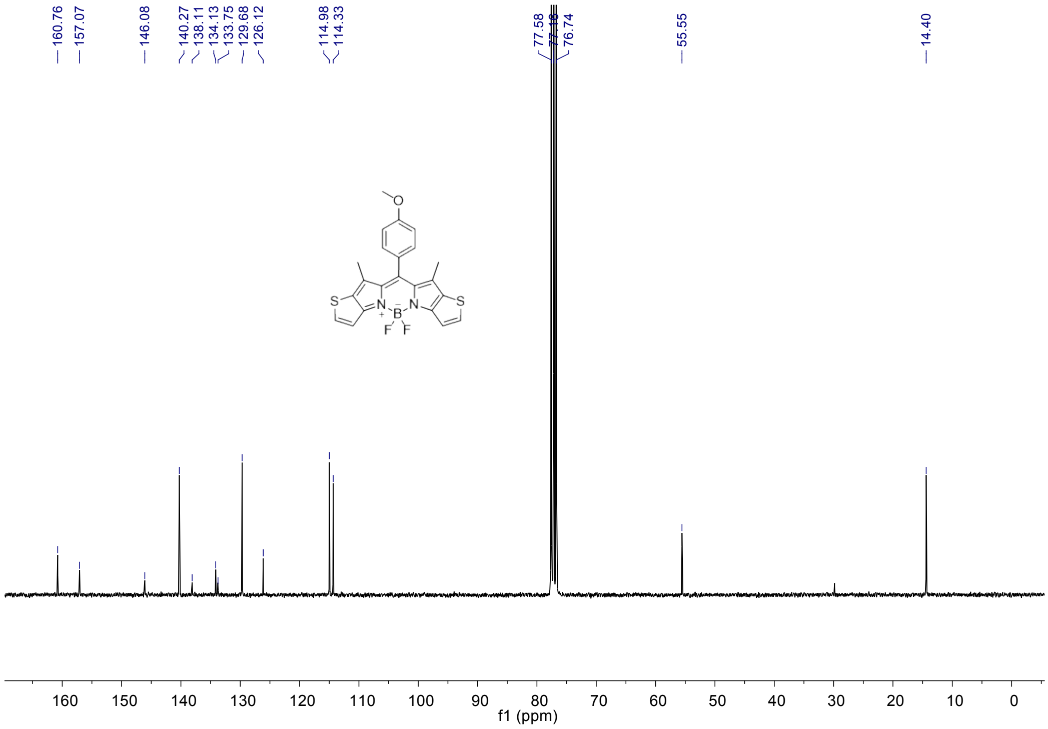

Fig. S10 ^{13}C NMR (75 MHz, ($CDCl_3$) of **MeO -BOD**.

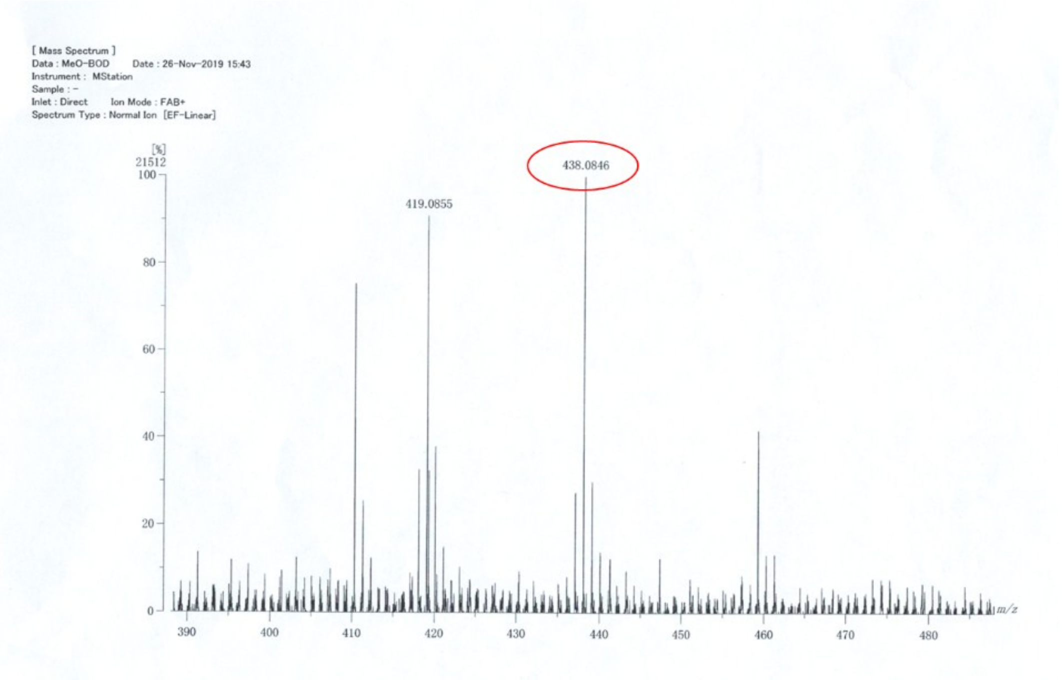

Fig. S11 High resolution mass spectrum of **MeO -BOD**.

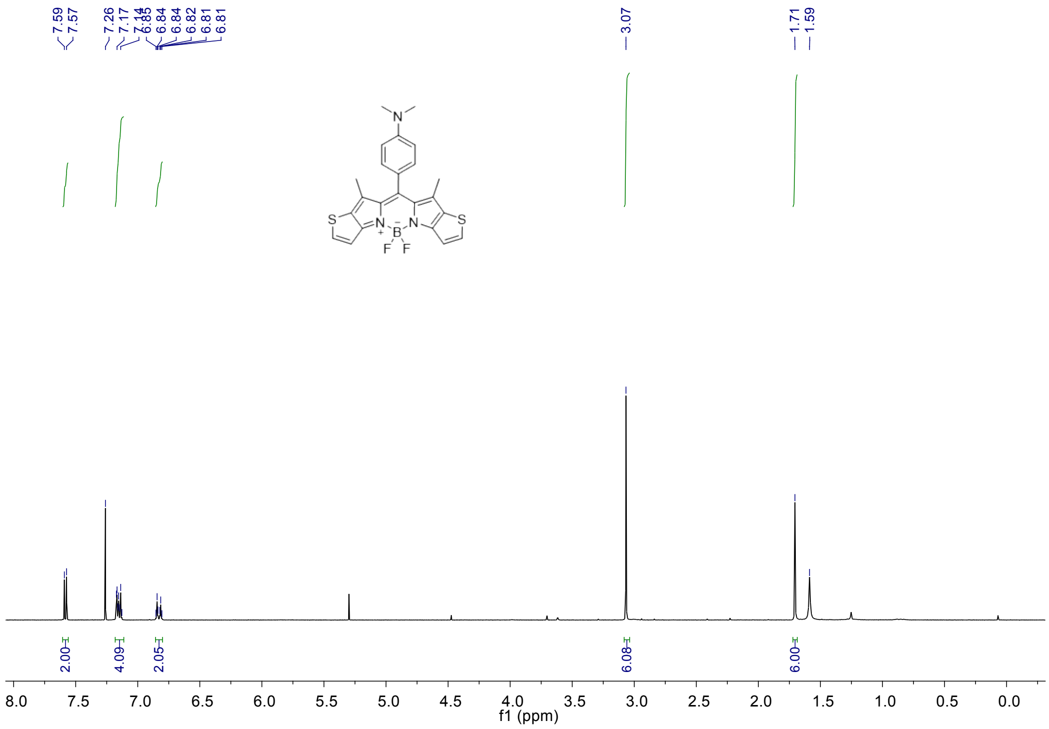

Fig. S12 ^{1}H NMR (300 MHz, $CDCl_3$) of **DMA-BOD**.

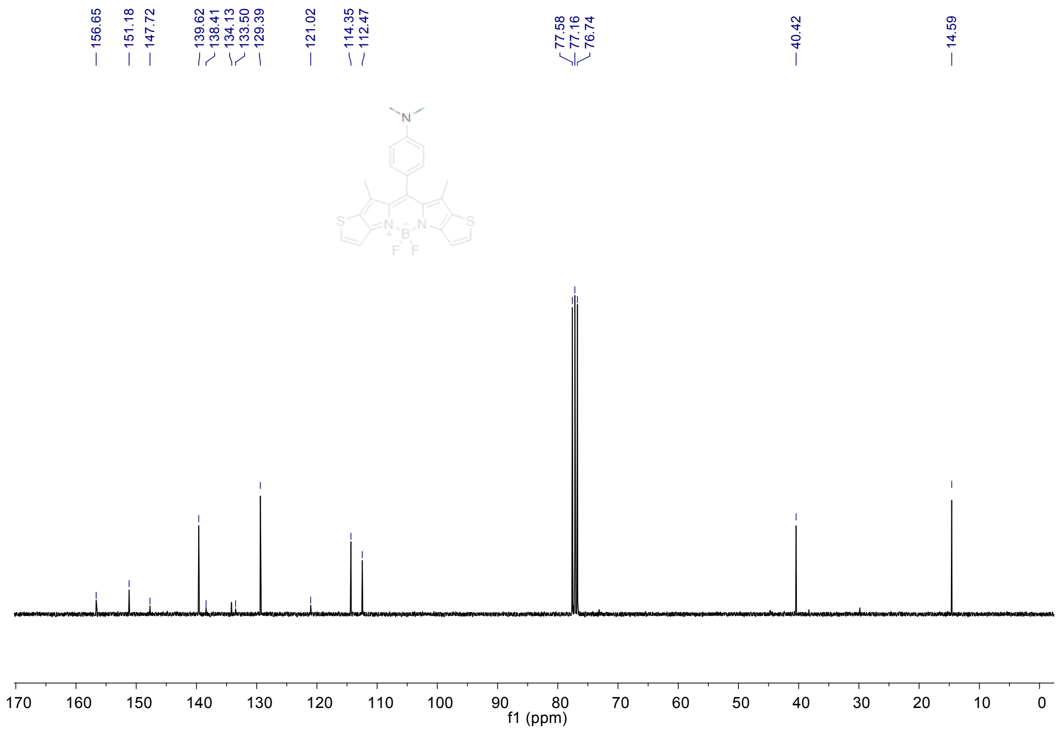

Fig. S13 ^{13}C NMR (75 MHz, $CDCl_3$) of **DMA-BOD**.

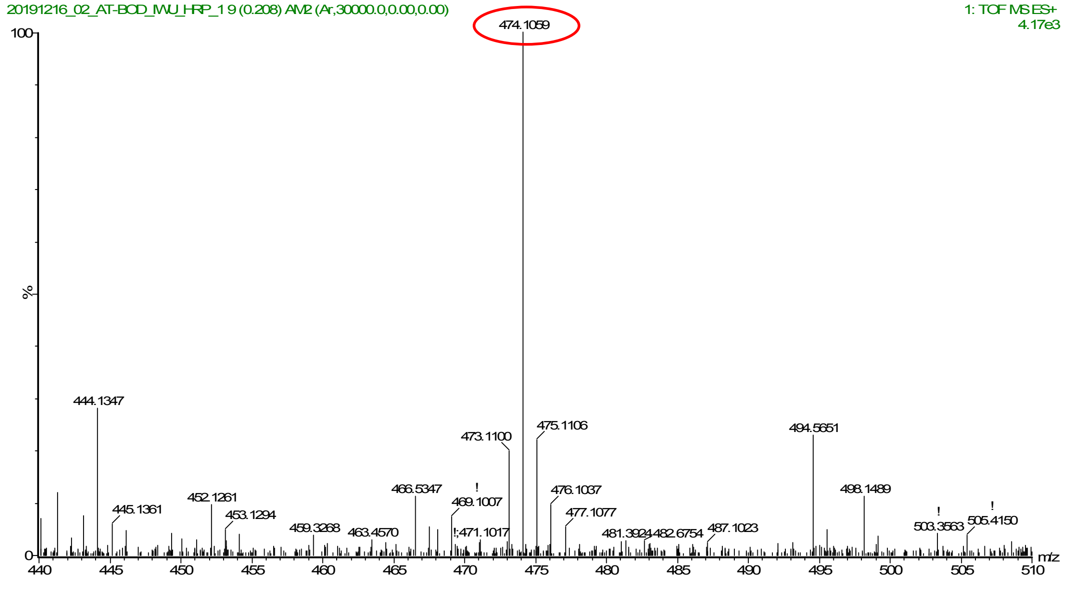

Fig. S14 High resolution mass spectrum of **DMA-BOD**.

4. Optical properties and biological studies

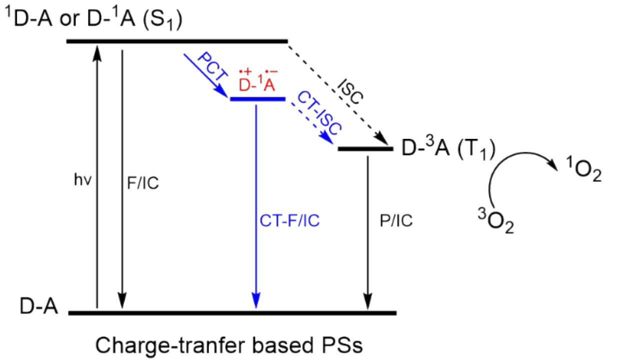

Scheme S1 The mechanism for charge-transfer based photosensitizers (PSs). D: donor; A: acceptor; F: fluorescence; IC: internal conversion; PCT: photoinduced charge transfer; CT: charge transfer; ISC: intersystem crossing; P: phosphorescence; S_1: first singlet excited state; T_1: first triplet excited state.

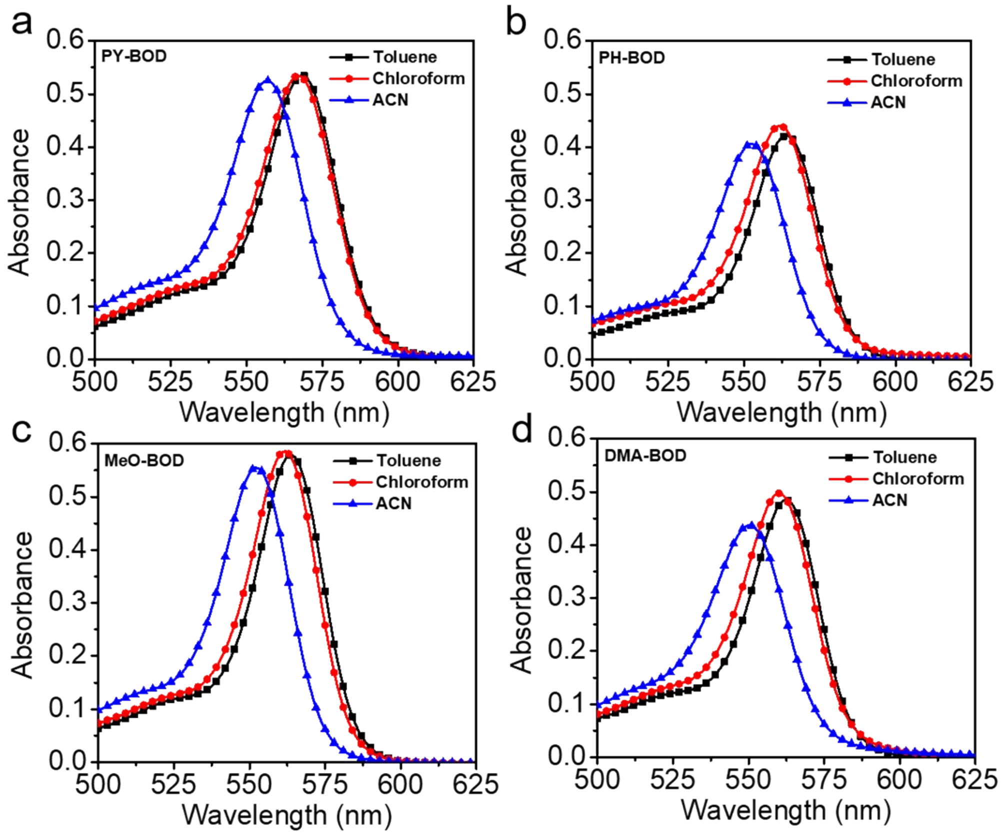

Fig. S15 UV-Vis absorption of (a) **PY-BOD**, (b) **PH-BOD**, (c) **MeO-BOD** and (d) **DMA-BOD**, in toluene (black), chloroform (red), CH_3CN (blue). c = 5.0 μM.

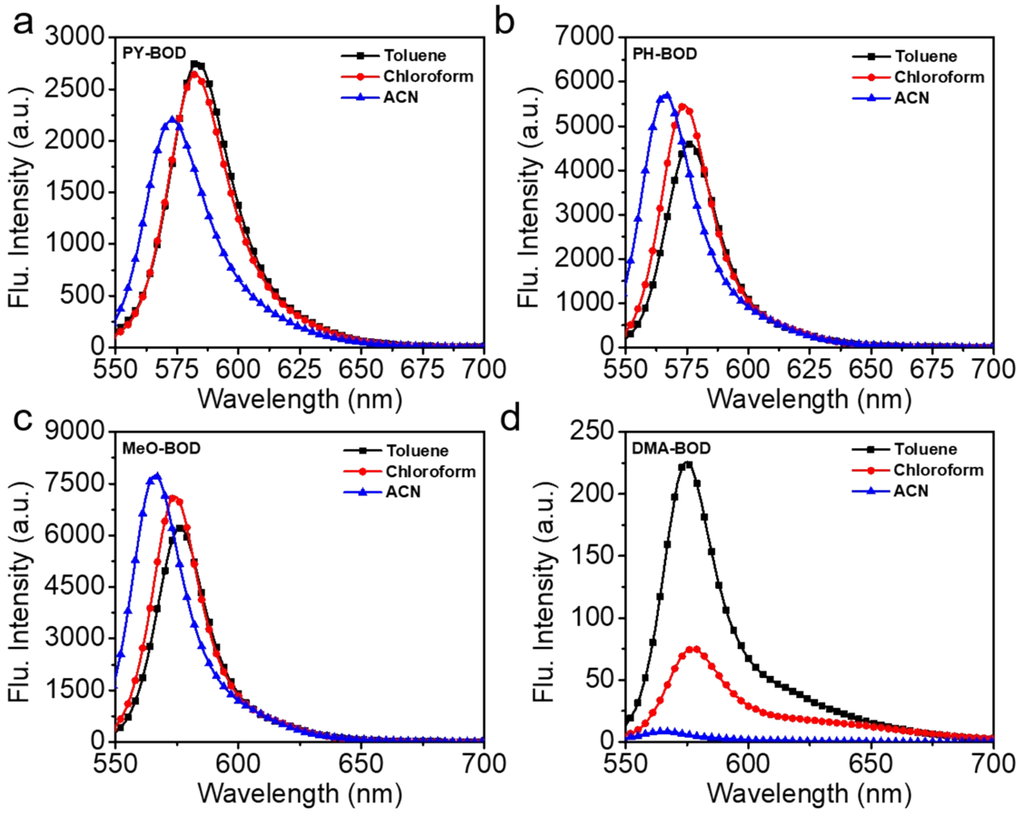

Fig. S16 The emission spectra of (a) **PY-BOD**, (b) **PH-BOD**, (c) **MeO-BOD** and (d) **DMA-BOD**, in toluene (black), chloroform (red), ACN (blue). c = 5.0 μM, λ_{ex} = 540 nm.

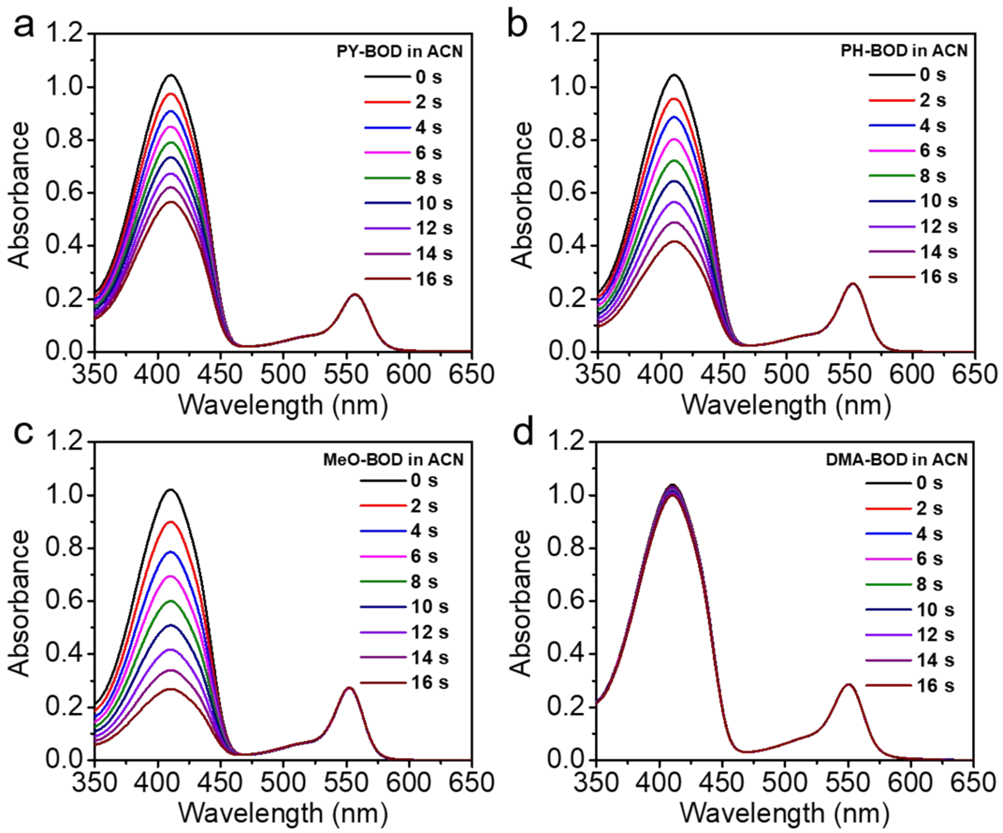

Fig. S17 Time-dependent photodegradation of DPBF with (a) **PY-BOD**, (b) **PH-BOD**, (c) **MeO-BOD** and (d**) DMA-BOD** in ACN under 560 nm light irradiation (0.1 W/cm^2).

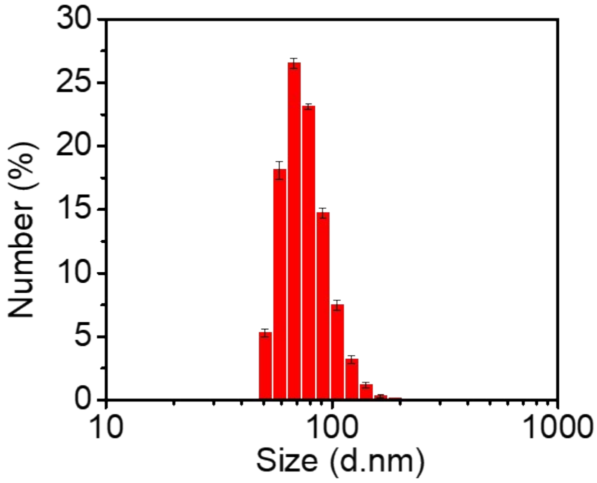

Fig. S18 Size distribution of **MeO-BOD** NPs in water detected by DLS.

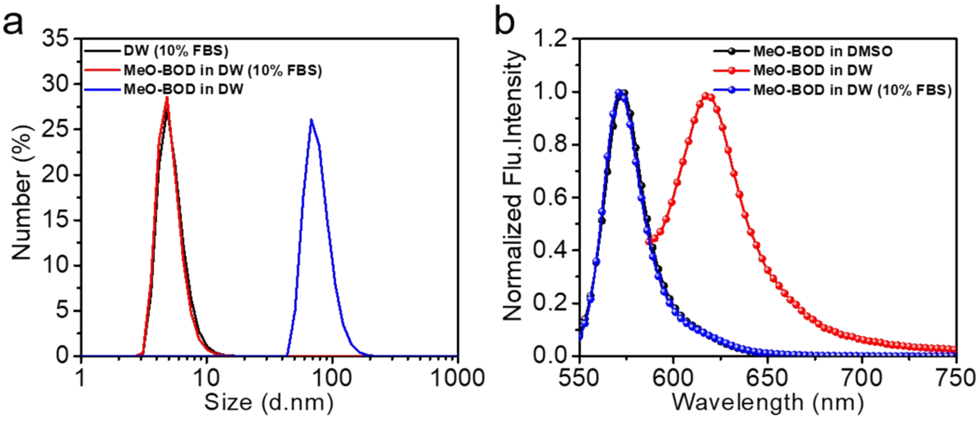

Fig. S19 (a) Size distribution of **MeO-BOD** in different medium detected by DLS. (b) fluorescence spectra of **MeO-BOD** in different solvents.

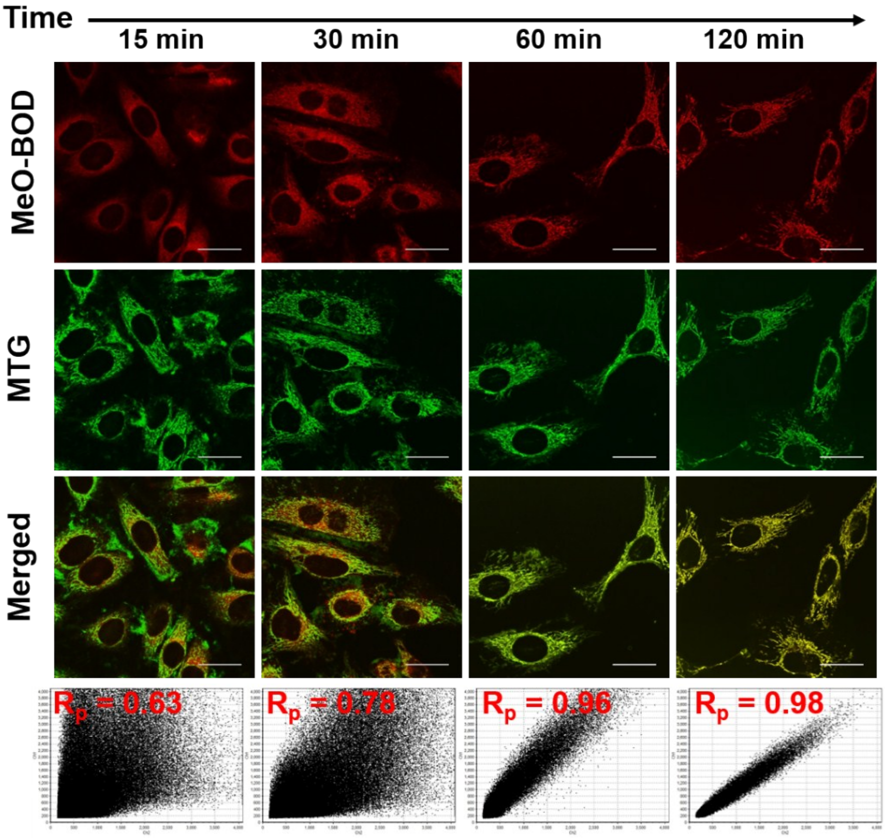

Fig. S20 Confocal laser scanning microscopy the time-dependent colocalization fluorescence images (15-120 min) of **MeO-BOD** (1.0 μM) with MTG (500 nM) in HeLa cells. R_p is Pearson's coefficient. Scale bar: 30 μm.

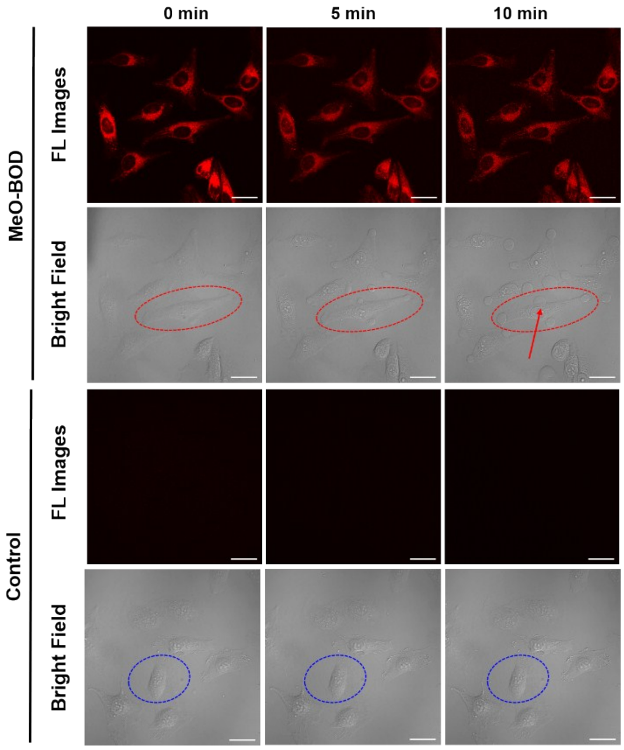

Fig. S21 Confocal laser scanning microscopy fluorescence images of HeLa cell with or without **MeO-BOD** (1.0 μM) and the time-dependent of bright field images (0-10 min) with 559 nm laser irritation (laser power 50%). The red arrow indicates membrane blebs. The red and blue dot arrows represent the cell membrane changes. Scale bar: 30 μm.

5. Supporting References

1 A. M. Brouwer, *Pure Appl. Chem.*, 2011, **83**, 2213-2228.

2 R. W. Redmond, J. N. Gamlin, *Photochem. Photobiol.*, 1999, **70**, 391-475.

3 M. Li, S. Long, Y. Kang, L. Guo, J. Wang, J. Fan, J. Du and X. Peng, *J. Am. Chem. Soc.*, 2018, **140**, 15820-15826.
